# Selection of Probiotics in the Prevention of Respiratory Tract Infections and Their Impact on Occupational Health: Scoping Review

**DOI:** 10.3390/nu13124419

**Published:** 2021-12-10

**Authors:** José Antonio Picó-Monllor, Beatriz Ruzafa-Costas, Eva Núñez-Delegido, Pedro Sánchez-Pellicer, Javier Peris-Berraco, Vicente Navarro-Lopez

**Affiliations:** 1 Department of Pharmacology, Pediatrics and Organic Chemistry, Faculty of Pharmacy, Universidad Miguel Hernández de Elche, 03202 Elche, Spain; 2 MiBioPath Research Group, Health and Science Faculty, Catholic University of Murcia (UCAM), Campus de los Jerónimos n 135, 30107 Murcia, Spain; beatriz.ruzafa@bioithas.com (B.R.-C.); eva.nunez@bioithas.com (E.N.-D.); pedro.sanchez@bioithas.com (P.S.-P.); vnavarro@ucam.edu (V.N.-L.); 3 Department R&D, Korott, s.l., 03801 Alcoy, Spain; jperis@korott.com; 4 Clinical Microbiology and Infectious Disease Unit, Hospital Universitario Vinalopó, 03293 Elche, Spain

**Keywords:** occupational health, probiotics, respiratory tract infections

## Abstract

The occupational health impact of respiratory infectious diseases is costly to the economy and the health care system. Probiotics are non-pathogenic live microorganisms that, when ingested in adequate amounts, can colonize the intestinal tract, and enhance the immune system. In recent years, numerous studies have described the possible usefulness of certain probiotic strains in the treatment and prevention of respiratory tract infections, with disparate results. In order to assess the possible efficacy and safety of these microorganisms to prevent or ameliorate respiratory tract infections, we systematically searched the bibliographic databases MEDLINE (via Pubmed), EMBASE, The Cochrane library, Scopus, and Web of science, using the descriptors “Respiratory Tract Infections”, “Probiotics”, “Occupational Health”, “Humans”, and “Clinical Trials”. After applying our inclusion and exclusion criteria, 18 studies were accepted for review and critical analysis. Our analysis suggests that a combination of different probiotics, most of them in the genus *Bifidobacterium* sp. and *Lactobacillus* sp., could be a good mix to strengthen the immune system and reduce the symptoms of URTIs in the healthy working population.

## 1. Introduction

According to the WHO, occupational health is an area of public health work whose mission is to promote and maintain the highest degree of physical, mental, and social well-being of workers in all occupations. Work-related health problems represent an economic loss of 4–6% of gross domestic product (GDP) in most countries [1].

Occupational health and safety contribute to reducing the costs of medical care, sick leave, and compensation for disability as they help avert the interruption of production processes and prevent occupational accidents and occupational diseases, as well as reducing labor passivity and absenteeism [2,3].

Chronic respiratory diseases, musculoskeletal disorders, noise-induced hearing loss, and skin problems are the most common occupational diseases. However, only a third of countries have programs to address these problems [1,4].

In 2006 in Beijing (Republic of China), the Global Alliance Against Chronic Respiratory Diseases (GARD) was created, led by the WHO. In its report on respiratory diseases and their impact worldwide, GARD describes the five respiratory diseases (chronic obstructive pulmonary disease, asthma, tuberculosis, lung cancer, and acute lower respiratory infections) that are among the most common causes of death all over the world. These are an immense health burden worldwide, and it is estimated that lower respiratory tract infections (LRTIs) cause almost 4 million deaths per year and are the leading cause of death among children under 5 years of age. Additionally, acute LRTIs in children set the stage for chronic respiratory diseases later in life. Moreover, those caused by the flu (influenza), kill 250,000 to 500,000 people and cost between USD 71 to 167 billion annually [5].

With these data, in 2017 the International Forum of Respiratory Societies (IFRS) wrote a report that exposes the tremendous impact that respiratory diseases have on world health and recommends, among other aspects, increasing research on the development of programs, tools, and strategies to prevent and better treat respiratory diseases by advocating for governmental (WHO) and non-governmental research organizations. Additionally, the IFRS promotes universal access to quality health care, including the availability of affordable essential medicines of guaranteed quality [6].

### 1.1. Respiratory Tract Infections

Respiratory tract infections (RTIs) owe their ethology to different viruses, such as rhinovirus, respiratory syncytial virus, influenza virus, human parainfluenza virus, human metapneumovirus, paramyxovirus, mycovirus, adenovirus and coronavirus, or bacteria such as *Streptococcus pneumoniae*, *Mycoplasma pneumoniae*, *Haemophiles influenzae*, *Chlamydia pneumoniae*, *Coxiella burnetii*, and *Legionella pneumophila*.

Infections of the respiratory system can affect the upper respiratory tract, which includes the nose, the paranasal sinuses, the pharynx, and larynx, as well as the lower respiratory tract, formed by the trachea, the bronchi, the bronchioles, and lungs. Upper respiratory tract infections (URTIs) cause tonsillitis, pharyngitis, sinus laryngitis, otitis media, certain types of flu, and the common cold, whereas LRTIs lead to more serious diseases such as pneumonia [7,8].

### 1.2. Probiotics

Probiotics are live microorganisms that, when administered in adequate amounts, confer a health benefit on the host [9]. Recently, the International Scientific Association for Probiotics & Prebiotics [10] established a consensus document with a series of criteria for microorganisms that are components of products aimed at the consumer to be considered probiotics:

- The microorganism in question must have been scientifically proven to be a safe species that is supported by sufficient evidence of general beneficial effect in humans.
- Evidence of its viability as a microorganism should be available in human studies conducted.

Recent meta-analyses and systematic reviews support the health benefits of probiotics in relation to the modulatory effect on the immune system, as reported in the study by Miller et al. [11] showing positive results in cellular immune function responsible for first-line defense against pathogenic microorganisms, or the work by Hajavi et al. [12], in which probiotics could increase systemic interferon (IFN), interleukin 10 (IL-10), and interleukin 12 (IL-12) production, elevate pro-Th1 cytokine immune responses, and decrease the profile of elevated Th2 cytokines in allergic diseases.

Darbandi et al. [8] and Hao et al. [13] in their studies suggest that probiotics are a complementary treatment in diseases of the respiratory system and a viable option to promote a faster recovery from such diseases. On the other hand, the study by Mahooti et al. [14], suggests that, due to the antiviral properties of probiotics against other viruses, they could be a complementary treatment alternative against SARS CoV-2.

In summary, supplementation with probiotics, prebiotics, and synbiotics has shown promising results against several enteric pathogens due to their unique ability to compete with the pathogenic microbiota, to kill it or to stimulate, modulate, and regulate the immune response of the host by initiating the activation of specific genes within and outside the intestinal tract of the host [15,16].

By virtue of their benefits, probiotic products can contribute significantly to the health of the population and, therefore, generate less expenditure for health systems. The works of Lenoir-Wijnkoop et al. [17,18] conducted in France and the USA, respectively, evaluated the impact on public health and the cost of the use of probiotics in RTIs. In the French study, it was found that the widespread use of probiotics could eliminate the equivalent of 2.4 million days of RTIs, 581,000 days off work, and 291,000 antibiotic treatments, which would generate health savings of between EUR 14.6 and 37.7 million per year. The study in the USA presented similar conclusions.

There are other systematic reviews and meta-analyses in which the results show a positive association between synbiotic [7] or probiotic [8,13,14] consumption and the prevention of RTIs in different populations. However, we have not found reviews in which this association is made with specific probiotic strains in a healthy working population.

Considering the benefits of certain probiotics in diseases of the respiratory system and their ability to stimulate the immune response of the host, we proposed this scoping review to identify and select potential probiotic strains to prevent URTIs, decrease the severity in those cases that end up developing the disease, and diminish their impact on occupational health.

## 2. Materials and Methods

### 2.1. Design

A descriptive study and critical analysis of works retrieved by scoping review according to Preferred Reporting Items for Systematic reviews and Meta-Analyses (PRIS-MA.

### 2.2. Data Collection Source

This review aimed to carry out a critical and systematic study of the works published in different databases through direct consultation and access via the Internet to works collected in the following databases: MEDLINE (via PubMed), EMBASE, SCOPUS, Cochrane Library Plus, and Institute for Scientific Information (ISI)-Web of Science.

### 2.3. Information Processing

To define the documentary search, the Thesaurus developed by the US National Library of Medicine (Medical Subjects Headings-Mesh) was used. Entry terms were also used. The terms “Occupational health”, “Probiotics”, and “Respiratory tract Infections” were used as descriptors and free text in titles and abstracts. The final search equation was developed for use in the MEDLINE database, via PubMed, by using the Boolean connectors and the “Humans” and “Clinical Trial” filters, with the following results:

(“Respiratory Tract Infections” [Title/Abstract] OR “Respiratory Infections” [Title/Abstract] OR ”Upper Respiratory Infections” [Title/Abstract] OR “Respiratory Tract Infections” [MeSH Terms]) AND (“Microbiota” [MeSH Terms] OR “microbiota*” [Title/Abstract] OR “Microbial Community” [Title/Abstract] OR “Microbial Communities” [Title/Abstract] OR “microbiome*” [Title/Abstract] OR “Microbial Flora” [Title/Abstract] OR “Microflora” [Title/Abstract] OR “Dysbiosis” [MeSH Terms] OR “Dysbiosis” [Title/Abstract] OR “Disbiosis” [Title/Abstract] OR “Dysbacteriosis” [Title/Abstract] OR “Probiotics” [MeSH Terms] OR “Probiotics” [Title/Abstract] OR “Dietary Supplements” [Title/Abstract]) OR “Occupational Health” [Mesh] OR “Occupational Health” [Title/Abstract] OR “Industrial Hygiene” [Title/Abstract] OR “Industrial Health” [Title/Abstract] OR “Occupational Safety” [Title/Abstract] OR “Employee Health” [Title/Abstract] OR “Occupational Risks” [Title/Abstract] OR “Insecure Labor Conditions” [Title/Abstract] OR “Occupational Risk” [Title/Abstract] OR “Work Risk” [Title/Abstract] OR “Occupational Hazard” [Title/Abstract] OR “Risk at Work” [Title/Abstract] OR “Professional Health” [Title/Abstract] OR “Working Conditions” [Title/Abstract] OR “Occupational Stress” [Mesh] OR “Job Stress” [Title/Abstract] OR “Professional Stress” [Title/Abstract] OR “Work Place Stress” [Title/Abstract] OR “Workplace Stress” [Title/Abstract] OR “Work Medicine” [Title/Abstract].

According to the different characteristics of the rest of the databases mentioned, the same strategy was adopted. The search was carried out from the first available date, until August 2021 (time of the last update). Additionally, as a secondary search and to reduce the number of papers not retrieved, the bibliographic list of articles that were selected in the main search was examined, in order to identify studies not detected in the review.

### 2.4. Final Selection of Articles

The final selection of the articles was made based on the following inclusion criteria: The papers had to be original clinical studies published in peer-reviewed journals, show a causal relationship between the intake of “probiotics” in “healthy subjects of a working age” and “respiratory tract infections”, selecting those pertinent whose full text could be retrieved, and be written in English, Portuguese, or Spanish. Those not carried out in humans or that did not focus the intervention on probiotics, on healthy people over 19 years old, and/or on RTIs were excluded.

The selection of the relevant articles was carried out independently by the authors of the present review (J.A.P.-M., B.R.-C., P.S.-P., E.N.-D., J.P.-B. and V.N.-L). To validate the choice of articles for the review, it was established that the assessment of concordance between two of these authors (Kappa index) should be greater than 0.80 (a measure of the strength of a very good agreement). Provided that this condition was met, possible disagreements would be resolved by consulting an expert in the field and subsequent consensus among the authors [19].

### 2.5. Assessment of Methodological Quality

The quality of the selected articles was assessed jointly, taking as support the guidelines for the communication of clinical studies, Consolidated Standards of Reporting Trials (CONSORT) [20], which contains a list of 25 essential points that should be described in the publication of these studies. For each selected article, a point was assigned for each item depending on whether the information was (“1”) or was not (“0”) collected in the article. In case the evaluation of any item was not necessary, that point was not counted in the total (not applicable = NA). When an item was composed of several points, these were evaluated independently, giving the same value to each of them, and later an average was calculated (this being the result for that item), in such a way that in no case could the score per item exceed 1 point.

### 2.6. Data Extraction

The control of the information extracted from the reviewed studies was carried out by means of double-entry tables that allowed the detection of errors and correction by re-consulting the originals. 

To determine the validity of the articles, the Burton–Kebler half-period (the median age) and the Price index (percentage of articles aged less than 5 years) were calculated. The articles were grouped according to the variables under study, to systematize and facilitate the understanding of the results, coding the following data: first author of the bibliographic reference and year of publication, study design, country where the study was carried out, number of participants, study population, period in which the work was carried out, what type of intervention took place, and the results obtained.

## 3. Results

**Figure 1:**
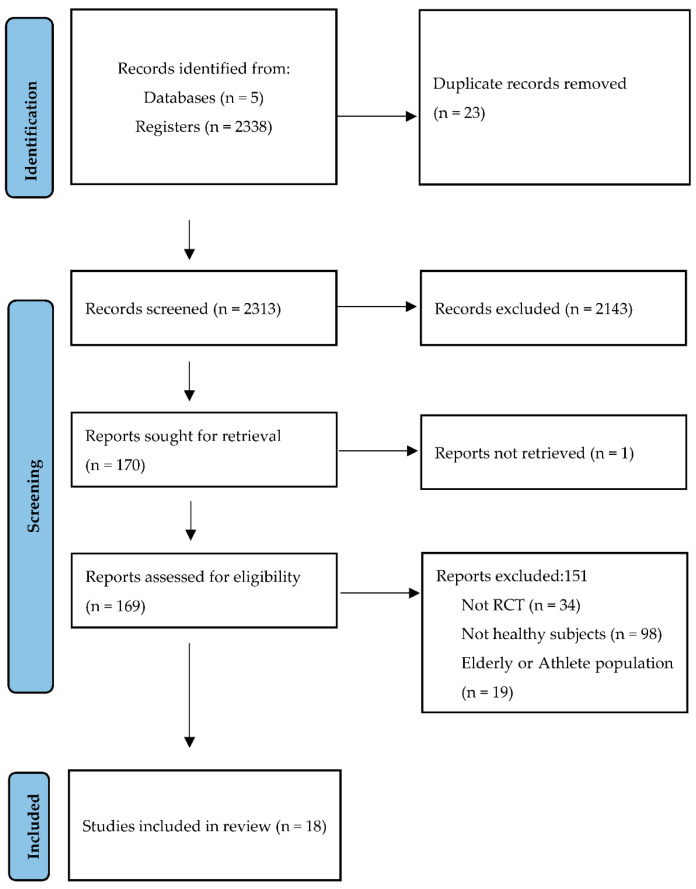
Identification and selection of studies according to Preferred Reporting Items for Systematic Reviews and Meta-Analyses (PRISMA statement) [21].

Using the search criteria described, 2338 references were retrieved: 1285 (54.96%) in MEDLINE, 6 in EMBASE (0.26%), 5 in Scopus (0.21%), 314 in the Cochrane Library (13.4%), and 728 in Web of Science (31.1%). After cleaning the duplicates, applying the inclusion and exclusion criteria, and consulting the bibliographic lists of the selected articles (Figure 1), it was possible to select 18 documents for review and critical analysis (Table 1).

The agreement on the relevance of the selected studies among all evaluators, calculated using the Kappa index, was 81%.

The chosen articles presented an obsolescence, according to the Burton–Kebler Index (IBK), equal to 6 years, with a Price Index (IP) of 38.9%. Pearson’s coefficient was r^2^ = 0.06. The years with the highest number of published works were 2021 with three [22,23,24] followed by two studies each in 2019 [25,26], 2018 [27,28], 2015 [29,30], 2011 [31,32], 2010 [33,34], and 2005 [35,36]. The years with only one publication were 2016 [37], 2012 [38], and 2013 [39].

When performing the CONSORT questionnaire, the scores ranged from a minimum of 13 (out of 25 items) to a maximum of 21.5 (out of 21 items), with a median equal to 18.5 (Table 2).

Most of the studies (88%) included in the review were double-blind clinical trials with placebo and control group [22,23,24,25,26,27,28,29,31,32,33,34,35,36,37,38,39], except for Shida et al. [30] without masking patients and researchers. Of these works, four were developed in Germany [22,29,33,36], three each in China [23,24,27] and the United States [32,37,38], two each in Malaysia [25,28], Japan [26,30], and Sweden [34,35], and a single study each in the countries of Denmark [29], Australia [39], and Italy [31]. All of them written in English.

According to the number of participants, the studies that presented the smallest sample size were Meng et al. [37] and Davidson et al. [32] with 30 and 40 people, respectively, while the works that presented the largest sample sizes were those of Guillemard et al. [33] and Ahrén et al. [22], with 1000 and 898 participants, respectively.

The population of the 18 studies comprised healthy people of both sexes and of working age always greater than 19 years [22,23,24,25,27,28,29,31,32,33,34,35,36,37,38,39], except for the works of Kinoshita et al. [26], which focused on women, exclusively, Shida et al. [30], which focused on men, exclusively, and Hor et al. [28], which studied an elderly population group. The follow-up periods of the studies included in this review ranged from a minimum of 4 weeks [37] to a maximum of 3 years [22].

Most of the selected studies assess the intake of different specific strains of probiotics in the healthy working population and their positive relationship in URTIs. The work of Zhang et al. [23], used a yogurt (Qingrun) with a mixture of *Bifidobacterium animalis* subsp. *lactis* Bl-04^®^, *Lacticaseibacillus casei*, *Lactobacillus delbrueckii bulgaricus*, and *Streptococcus thermophilus* with two plant species used in traditional Chinese medicine, *Eriobotrya japonica* and *Pyrus nivalis*.

The species and strains used in the interventions were all non-sporulated Gram-positive bacteria, and the concentrations ranged between 10^6^ and 10^11^ colony-forming units (CFU). There is a current reclassification of the generic term Lactobacilli that reflects the phylogenetic position of microorganisms, and groups lactobacilli into robust clades with shared ecological and metabolic properties [40]. In this way, *Lactobacillus plantarum* was renamed *Lactiplantibacillus plantarum* (*L. plantarum*); *Lactobacillus paracasei* subsp. *paracasei* was renamed *Lacticaseibacillus paracasei* subsp. *paracasei* (*L. paracasei* subsp. *paracasei*); *Lactobacillus fermentum* was renamed *Limosilactobacillus fermentum* (*L. fermentum*); *Lactobacillus casei* was renamed *Lacticaseibacillus casei* (*L. casei*); *Lactobacillus rhamnosus* was renamed *Lacticaseibacillus rhamnosus* (*L. rhamnosus*); and *Lactobacillus reuteri* was renamed *Limosilactobacillus reuteri* (*L. reuteri*).

The different probiotics used in the 18 studies were: *L. plantarum* HEAL9, *L. paracasei* subsp. *paracasei* 8700:2, *Streptococcus thermophilus* ENT-K12, *L. plantarum* DR7, *Lactobacillus delbrueckii bulgaricus* OLL1073R-1, *Streptococcus thermophilus*, *L. paracasei* subsp. *paracasei*, *L. casei* 431^®^, *L. fermentum* PCC^®^, *L. casei* Zhang, *Bifidobacterium animalis* subsp. *lactis* BB-12^®^, *L. casei* Shirota^®^, L. *rhamnosus* LGG^®^, *Bifidobacterium animalis* subsp. *lactis* Bl-04^®^, *Bifidobacterium animalis* subsp. *lactis* Bi-07^®^; *Lactobacillus acidophilus* NCFM^®^; *L. rhamnosus* LGG^®^; Verum (*L. casei* DN-114 001, *Lactobacillus delbrueckii bulgaricus* and *Streptococcus thermophilus*); *Lactobacillus delbrueckii* subsp. *bulgaricus*; Tribion harmonis™ (*Lactobacillus gasseri* PA 16/8, *Bifidobacterium longum* SP 07/3, *Bifidobacterium bifidum* MF 20/5), and *L. reuteri* protectis (ATCC55730).

The most used bacterial species, individually or in combination, were *B. animalis* subsp *lactis* and *L. casei* on six occasions and *L. plantarum*, *L. paracasei*, and *L. delbrueckii* subsp. *bulgaricus* on three occasions each.

In addition to the interventions with these different strains of probiotics, three of the selected studies administered flu vaccines [29,31,32].

In the selected studies, the intake of different probiotic strains in healthy working populations and the impact on upper respiratory tract diseases were analyzed. The results of the different interventions were varied.

The works of Tubelius et al. [35], Berggren et al. [34], and De Vrese et al. [36] demonstrated a significant relationship (*p* < 0.05) between the oral intake of a mixture of probiotics (*L. plantarum* HEAL9, *L. paracasei* subsp. *paracasei* 8700: 2; Tribion harmonis™; *L. reuteri*) and a lower incidence of having one or more episodes of the common cold and fewer days of experiencing the symptoms of the common cold. In the study by Ahrén et al. [22] a positive association was demonstrated between two probiotic strains (*L. plantarum* HEAL9, *L. paracasei* subsp. *paracasei* 8700: 2) and protection against colds in adults prone to colds, but without reducing the severity or incidence of colds.

Zhang et al. [23] and Shida et al. [30] concluded that the probiotic mixture Qingrun and *L. casei* Shirota^®^, respectively, presented a protective effect against URTIs in office workers, reducing the incidence, duration, and severity of the same and improving the immune biomarkers. Recently, the results of Wang et al. [24] in their work on first-line medical personnel during the COVID-19 pandemic (medical staff) stand out. The ingestion of the *Streptococcus thermophilus* ENT-K12 strain produced a homeostatic relationship between the oropharyngeal microbiota and the cells of the immune system, for at least 20 days. Such a balance could protect medical personnel fighting RTIs, including COVID-19.

The works by Chong et al. [25] and Hor et al. [28] used a single strain, *L. plantarum* DR7 and *L. casei* Zhang, respectively, with similar results in improving the symptoms of URTIs, mainly through the induction of immunomodulatory and anti-inflammatory effects. Additionally, the studies by Guillemard et al. [33] and West et al. [39] significantly associated the incidence of common infections, including respiratory infections, with the ingestion of the probiotic *L. casei* DN-114 001 and *Bifidobacterium animalis* subsp. *lactis* Bl-04^®^, respectively. However, the results of the work of Kinoshita et al. [26] with a probiotic mixture of the strain OLL1073R-1 and *Streptococcus thermophilus* did not show any prevention of influenza or improvement in the activity of NK cells. However, they did observe a significant increase in the production of γ-interferon (IFN-γ).

Three studies, by Jespersen et al. [29], Rizzardini et al. [31], and Davidson et al. [32], with *L. paracasei* subsp. *paracasei* 431^®^ and *L. rhamnosus* LGG^®^ and *Bifidobacterium animalis* subsp. *lactis* BB-12^®^, in patients who received the influenza vaccine and live attenuated influenza vaccine (LAIV), presented different results. In the case of *L. paracasei* subsp. *paracasei* 431^®^, no improvement was noted in the immune response or the concentration of specific antibodies. However, the *L. rhamnosus* LGG^®^ strain and *Bifidobacterium animalis* subsp. *lactis* BB-12^®^, did improve the immunogenicity of the vaccine and, in addition, the mixture of these two strains in the work of Smith et al. [38] presented a positive association with the Health-Related Quality of Life (HRQoL) perceived by a group of healthy students during the symptoms of URTIs. The results of the work of Meng et al. [37], with the probiotic strain *Bifidobacterium animalis* subsp. *lactis* BB-12^®^, also had a positive impact on the immune system and the severity of URTIs.

## 4. Discussion

The study of the novelty or obsolescence of the chosen topic is quite valid and interesting, since, of all of articles retrieved, approximately 40% were published in the last 5 years. The Burton Kebler index presented a value in accordance with the expected, while the Price index obtained a slightly higher value in health sciences. However, the Pearson coefficient indicated that the selected articles presented a non-statistically significant increasing linear regression model (*p* = 0.33) [41].

On the other hand, according to the degree of evidence and recommendations of the US Preventive Services Task Force (USPSTF) [42], controlled and randomized clinical trials (RCTs) are those that provide the most scientific evidence due to their consistent cause–effect relationships. The assessment of the quality of the studies included in this review using CONSORT was acceptable, with a mean of 18.5 out of 25. Therefore, the grade of recommendation was B (moderate evidence that the measure is effective, and the benefits outweigh the harm).

Likewise, English was the language chosen for the publication of most of the articles since publication in a different language is negative for the impact factor and citations [43]. Furthermore, the number of journals written in English contained in the databases is currently very high [44].

The 18 selected studies focused on URTIs and were carried out on a healthy, working population of men and women, except for the study by Shida et al. [30], which focused exclusively on men, and the work by Kinoshita et al., which was focused exclusively on women. Reinforcing the use of probiotics in other population groups to improve or prevent URTIs, were the works of Makino et al. [45], Shinkai et al. [46] in the elderly population, Wilcox et al. [47] in adults and children, and the study by Tavares-Silva et al. [48] in marathon runners. All probiotic species in the studies analyzed are classified as QPS (qualified presumption of safety). There were hardly any adverse effects in the populations tested.

The fact that in the studies analyzed in this scoping review, 15 different strains of bacteria were used successfully, either alone or in different combinations, suggests that not only that one specific strain or a single combination of probiotics will work, but that many combinations could be a good treatment option. To select the appropriate combination of strains, important factors must be considered, such as the fact that not all combinations can obtain positive results. In this sense, the association of *Lactobacillus delbrueckii bulgaricus* OLL1073R-1 and *Streptococcus thermophilus* [26] did not show a significant preventive effect against influenza or enhancement in NK lymphocyte activity. It is evident that, in a healthy working population, there are variations between strains in terms of their immunomodulatory capacity on the immune system [22,23,25,27,28,29,30,31,32,33,34,36,37]. The dose used, the duration of the intervention, and seasonal factors may also influence the interpretation of the results [22,23,24,25,26,27,28,29,30,31,32,33,34,35,36,37,38,39].

In the selected studies, we observed that the genus *Lactobacillus* [22,23,25,26,27,28,29,30,31,32,33,34,35,36,38] predominated against *Bifidobacterium* [23,31,36,37,38,39]. The combination of *L. plantarum* HEAL9 and *L. paracasei* subsp. *paracasei* 8700:2 [22,34] and the mixture of *L. paracasei* subsp. *paracasei*, *L. casei* 431^®^, and *L. fermentum* PCC^®^ [27] presented statistically significant favorable results for the biomarker IFN-γ (*p* = 0.045) and IFN-γ (*p* < 0.001) respectively compared to the placebo group, and in reducing the incidence of URTIs.

Six strains of the genus *Lactobacillus*, namely *L. plantarum* DR7, *L. casei* Zhang, *L. casei* 431^®^, *L. casei* Shirota^®^, *L. rhamnosus* LGG^®^, and *L. reuteri* protectis (ATCC55730), have been used successfully alone in decreasing the risk of contracting URTIs and duration of the symptoms of the common cold [25,28,29,30,35] as well as in increasing the immunogenicity of an intranasal vaccine against influenza (LAIV) [32]. In agreement with these results are the works of Fonolla et al. [49], Namba et al. [50], and Atkasu et al. [51], although they used different probiotic strains on the elderly population. According to a review by Li et al. [52], which highlights that probiotics induce cell-mediated immunity in phagocytes and natural killer (NK) cells and promote IgA secretion in saliva to enhance vaccine effects, the mechanisms of probiotics in terms of their effects on immune function may be varied. Furthermore, probiotic metabolites, such as short-chain fatty acids, and peptidoglycan components of probiotics appear beneficial to both the host intestinal epithelium and the microbiota by modulating immune function.

The genus *Bifidobacterium* in our scoping review was always administered in a multi-strain probiotic preparation with the genus *Lactobacillus* [27,31,36,37,38,39] or mixed with yogurt smoothies [37]. These combinations with other strains make it almost impossible to assess their specific contribution to the observed effects. However, *Bifidobacterium animalis* subsp. *lactis* BB-12^®^ and *Bifidobacterium animalis* subsp. *lactis* Bl-04^®^ [37,39] demonstrated by themselves a beneficial effect on immunity and reduction in the risk of URTIs in healthy workers.

We highlight the positive results shown with the oropharyngeal probiotic strain *Streptococcus thermophilus* ENT-K12 [24] used in medical personnel who were in close contact with patients hospitalized for COVID-19, including the formation of a stable microbiota in the oral cavity, protection from respiratory infections for at least 20 days, shorter duration of URTI symptoms, reduced days off due to sick leave, and less use of antibiotics and antivirals. These results should be interpreted with caution, however, because there were some limitations such as non-masking, a small sample size, little follow-up period for medical personnel, etc.

Regarding the use of probiotics in the prevention of COVID-19 in health workers, more studies are currently being carried out to contrast their effectiveness. These are studies that are currently recruiting patients and are registered in the American registry of clinical trials, clinicaltrials.gov (accessed on 4 October 2021) and identified with the codes NCT04366180 and NCT04462627.

The use of preparations with fermented dairy products, such as Qingrun, Verum^®^, and Tribion harmonis™, could suggest, based on the good results obtained in shift workers [33] and office workers [23], that the combination of strains and species with beneficial effects on the immune system could be a potential nutritional strategy that would address the global problem of respiratory infections and help decrease days off work due to the symptoms caused by URTIs. In accordance with this postulate, Chan et al. [7] in their meta-analysis “Preventing Respiratory Tract Infections by Synbiotic Interventions,” proposes a similar strategy for improvement of URTIs, in addition to mitigating the misuse of antibiotics used in their treatment.

### Limitations

The results of this review were limited by the shortcomings of each work reviewed [53]. Some commented on the study by Wang et al. [24] or the short follow-up period of 1 month [24,32,37] or the number of people enrolled, [32,37] which could indicate a lack of expected scientific rigor.

Another possible limitation was the small number of articles [22,23,24,25,26,27,28,29,30,31,32,33,34,35,36,37,38,39] that found and specifically developed an association between the intake of probiotics in working people and RTIs. Furthermore, the population was not homogeneous [28,38], and in some cases the follow-up of the population did not allow the interpretation of favorable results.

## 5. Conclusions

The results of the studies included in this review suggest that probiotics may be a therapeutic tool for public health in URTIs by improving the immune system and reducing days off work. There were practically no adverse effects in the populations tested.

Since probiotics appear to be candidates for the prevention of URTIs and generate significant health savings [17,18], and taking into account the above information, we suggest that a combination including some the probiotics (*L. plantarum* HEAL9, *L. paracasei* subsp. *paracasei* 8700: 2, *L. plantarum* DR7, *L. casei* Zhang, *L. casei* 431^®^, *L. casei* Shirota^®^, *L. rhamnosus* LGG^®^, *Lactobacillus gasseri* PA 16/8, *L. reuteri*, *Bifidobacterium animalis* subsp. *lactis* BB-12^®^ *Bifidobacterium animalis* subsp. *lactis* Bl-04^®^, *Bifidobacterium longum* SP 07/3, and *Bifidobacterium bifidum* MF 20/5) should be tested in healthy working populations through clinical trials to evaluate their effectiveness in preventing URTIs and identify the best dosage.

## Figures and Tables

**Table 1:** Summary of reviewed studies on the relationship between probiotics and respiratory tract infections in healthy subjects.

Author (Year)	Design	Country	Participants	Population	Monitoring	Intervention Performed	Results
Ahrén (2021) [22]	Randomized, double-blind, placebo-controlled trial multicentric	Germany	898 M/F: 322/576 Median age: 40.6 years	Healthy men and women	3 years	*Lactiplantibacillus plantarum* HEAL9 and *Lacticaseibacillus paracasei* 8700:2 (1 × 10^9^ cfu)	Protects against multiple colds in adults prone to getting colds.
Zhang (2021) [23]	Randomized, double-blinded placebo-controlled clinical trial	China	123 M/F: 69/54 Median age 37.2 years	Healthy white-collar workers	12 weeks	Qingrun yogurt with *Bifidobacterium animalis* subsp. *lactis* Bl-04 (2 × 10^9^ cfu), *Lactobacillus casei, Lactobacillus bulgaricus* and *Streptococcus thermophilus*	Qingrun yogurt was able to reduce the incidence, duration, and severity of URTIs and improve immune biomarkers.
Wang (2021) [24]	Randomized controlled clinical trial: Study pilot. Multicentric	China	193 M/F: 56/137 Median age: 36.1 years	Healthcare workers	1 month	*Streptococcus thermophilus* ENT-K12	ENT-K12 creates a stable URT microbiota for at least 20 days and protects medical staff from URTIs, can reduce the length of sick days, days off work, and days on antibiotics and antivirals.
Chong (2019) [25]	Randomized double-blind placebo-controlled study	Malaysia	109 Age: <30 and 30 to 60 years	Healthy men and women	12 weeks	*Lactobacillus plantarum* DR7	DR7 can protect adult populations against URTIs by inducing immunomodulatory and anti-inflammatory effects while promoting mucosal barrier integrity and actions of NK cells.
Kinoshita (2019) [26]	Randomized controlled open-label study	Japan	F: 961 Median age: 39.3 years	Women health workers	16 weeks	*Lactobacillus delbrueckii* subsp. *bulgaricus* (OLL1073R-1) and *Streptococcus thermophilus* (1 × 10^9^ cfu)	The probiotic mixture did not show any prevention of influenza and NK cell activity enhancement. However, a significant increase in IFN-γ production was found.
Zhang (2018) [27]	Randomized double-blind placebo-controlled prospective trial. Single center	China	134 M/F: 66/68 Median age: 34.3 years	Healthy subjects	12 weeks	*Lactobacillus paracasei* ≥ 3 × 10^7^ cfu, *L. casei* 431^®^ ≥ 3 × 10^7^ cfu and *Lactobacillus fermentum* PCC^®^ ≥ 3 × 10^6^ cfu	Mix probiotics were safe and effective for fighting the common cold and influenza-like respiratory infections by boosting the immune system.
Hor (2018) [28]	Randomized, double-blind and placebo-controlled study	Malaysia	137 M/F: 62/75 Median age: 44.2 years	Healthy adults and elderly	12 months	*Lactobacillus casei* Zhang (10^9^ cfu)	LCZ alleviated URTI symptoms in adults, with reduced duration for nasal, pharyngeal, general flu, and total respiratory illness symptoms compared to the placebo.
Meng (2016) [37]	Randomized, partially blinded, four-period crossover study	USA	30 M/F: 11/29 Median age: 28 years	Healthy subjects	4 weeks	(i) YS (ii) YS with BB-12^®^ added prefermentation (PRE); (iii) YS with BB-12^®^ added postfermentation (POST) (iv) one capsule BB-12^®^	The timing of BB-12 addition to yogurt smoothies in relation to the fermentation process influenced the impact of BB-12 on immune function and cold/flu severity in young healthy adults.
Jespersen (2015) [29]	Randomized double-blind, placebo-controlled trial multicentric	Denmark/Germany	1104 M/F: 453/651 Median age: 31.6 years	Healthy men and women with influenza vaccination	42 days	*L. casei* 431^®^ (≥10^9^ cfu)	*L. casei* 431 did not show an effect on antibody titers and influenza A–specific antibodies 3 weeks after influenza vaccination but may reduce the duration of common cold and ILI episodes in healthy adults.
Shida (2015) [30]	Randomized controlled trial	Japan	M: 100 Median age: 40.6 years	Healthy male workers	4 months	*Lactobacillus casei* Shirota^®^ (1.0 × 10^11^ cfu) fermented milk	Fermented milk with LcS may reduce the risk of URTIs in healthy middle-aged office workers.
Smith (2012) [38]	Randomized double blind placebo-controlled trial	USA	198 M/F: 47/151 Age: 19 to 25 years	Healthy subjects	12 weeks	*Lactobacillus rhamnosus* LGG^®^ and *Bifidobacterium animalis* ssp. *lactis* BB-12^®^ (1 × 10^9^ cfu)	Mix probiotics LGG^®^ and BB-12^®^ may be beneficial for mitigating decrements in HRQL during URTI in college students living on campus in residence halls.
West (2013) [39]	Randomized double-blind placebo-controlled parallel trial	Australia	465 M/F: 241/224 Median age: 36 years	Healthy subjects	160 days	*Bifidobacterium animalis* subsp. *lactis Bl-04* (2 × 10^9^ cfu), *Lactobacillus acidophilus NCFM* and *Bifidobacterium animalis* subsp. *lactis Bi-07 (NCFM & Bi-07*) (5 × 10^9^ cfu)	Bl-04 appears to be a useful nutritional supplement in reducing the risk of URTI in healthy, physically active adults.
Rizzardini (2011) [31]	Randomized, double-blind, placebo controlled, four-arm, parallel-group study	Italy	211 M/F: 93/118 Median age: 29 and 37.3 years	Healthy adults with influenza vaccination	6 weeks	BB-12^®^ capsule or *L. casei* 431^®^ drink (1 × 10^9^ cfu) and Fluad^®^	BB-12 or *L. casei* 431^®^ significantly increases antigen-specific immune responses in healthy individuals receiving an influenza vaccination.
Davidson (2011) [32]	Randomized double-blind placebo-controlled pilot study	USA	42 M/F: 16/26 Median age: 33.5 years	Healthy adults with LAIV	28 days	*Lactobacillus GG* (1 × 10^10^ cfu) and LAIV	*Lactobacillus* GG^®^: is potentially an important adjuvant to improve influenza vaccine immunogenicity.
Guillemard (2010) [33]	Randomized, double-blind, controlled study Single-center	Germany	1000 M/F: 220/780 Median age: 31.8 years	Healthy men and women	4 months and 2 weeks	Verum: *Lactobacillus casei* DN-114 001 (1 × 10^10^ cfu), *Streptococcus thermophilus*, *Lactobacillus delbrueckii* subsp. *bulgaricus*	Verum consumption was associated with significant improvement in incidence of CIDs in shift workers.
Berggren (2010) [34]	Randomized, double-blind, placebo-controlled trial multicentric	Sweden	272 M/F: 92/180 Median age: 46.5 years	Healthy men and women	12 weeks	*Lactiplantibacillus plantarum* HEAL9 and *Lacticaseibacillus paracasei* 8700:2 (1 × 10^9^) cfu	Contributes to the body’s defense against common cold infections *p* < 0.05.
De Vrese (2005) [36]	Randomized, double-blind, placebo-controlled intervention study	Germany	479 M/F: 185/294 Median age: 37 years	Healthy men and women	8.5 months	*Lactobacillus gasseri* PA 16/8, *Bifidobacterium longum* SP 07/3, *Bifidobacterium bifidum* MF 20/5 (5 × 10^7^ cfu) (Tribion harmonis^TM^)	The intake of mix probiotic for at least 3 months significantly shortened common cold episodes by almost 2 days and reduced the severity of symptoms.
Tubelius (2005) [35]	Randomized, double-blind placebo-controlled study	Sweden	181 M/F: 65/71 Median age: 44 years	Healthy workers	80 days	*Lactobacillus reuteri* protectis (ATCC55730) 1 × 10^8^ cfu	*Lactobacillus reuteri* is effective to promote workplace healthiness. In the studied population, sick-days caused by respiratory or gastrointestinal diseases could be reduced by 55%.

**M/F**: number males and females. **URTIs**: Upper Respiratory Tract Infections. **HEAL9**: *Lactiplantibacillus plantarum* specific strain. **8700:2**: *Lacticaseibacillus paracasei* specific strain. **ENT-K12**: *Streptococcus thermophilus* specific strain. **CFU**: Colony-forming units. **LCZ**: *Lactobacillus casei* Zhang. **NK**: Natural Killer cell. **IFN-γ**: Interferon gamma. **DR7**: *Lactobacillus plantarum* specific strain. **OLL1073R-1:** *Lactobacillus delbrueckii* subsp. *bulgaricus* specific strain. **431^®^**: *Lactobacillus paracasei* ssp. *paracasei* specific strain. **PCC^®^**: *Lactobacillus fermentum* specific strain. **ILI**: Influenza-Like Illness. **YS**: Yogurt smoothie. **HRQL**: Health Related Quality of Life. **LcS**: *Lactobacillus casei* Shirota^®^ specific strain. **LGG^®^**: *Lactobacillus rhamnosus*. specific strain. **Bl-04**: *Bifidobacterium animalis* subsp. *lactis* specific strain. **NCFM & Bi-07:** *Lactobacillus acidophilus and Bifidobacterium animalis* subsp. *lactis* specific strain. **BB-12^®^**: *Bifidobacterium animalis* subsp. *lactis* specific strain. **URT**: Upper Respiratory Tract. **Fluad^®^**: Influenza vaccine. **LAIV**: Live Attenuated Influenza Vaccine. **CDIs**: Common Infectious Diseases (respiratory and gastrointestinal). **Verum**: mix probiotics 1 × 10^10^ (*Lactobacillus casei* DN-114 001 *Streptococcus thermophilus and Lactobacillus delbrueckii* subsp. *Bulgaricus)*. **Tribion harmonis^TM^**: mix probiotics *Lactobacillus gasseri* PA 16/8, *Bifidobacterium longum* SP 07/3, *Bifidobacterium bifidum* MF 20/5 specific strains.

**Table 2:** Assessment of the methodological quality of the studies analyzed by means of the 25 items of the CONSORT 2010 [20].

	1	2	3	4	5	6	7	8	9	10	11	12	13	14	15	16	17	18	19	20	21	22	23	24	25	T	%
Ahrén [22]	1	1	1	1	1	1	0.5	1	0	1	1	1	1	0.5	1	1	0.5	NA	1	1	1	1	1	1	1	21.5	90
Zhang [23]	1	1	1	1	1	0.5	0	0	0	1	1	0.5	1	0.5	1	1	0.5	NA	0	1	1	1	1	1	1	18	75
Wang [24]	0.5	1	1	1	1	0.5	0	0	0	1	1	0.5	1	0.5	1	1	0.5	NA	1	1	1	1	1	1	1	18.5	77
Chong [25]	1	1	1	1	1	0.5	0.5	0.5	0	0	0	0.5	1	0.5	1	1	1	NA	1	0	1	1	0	0	1	15.5	65
Kinoshita [26]	1	1	1	1	1	0.5	0.5	1	1	1	1	0.5	1	0.5	1	1	0.5	NA	1	1	1	1	1	1	1	21.5	90
Zhang [27]	0.5	1	1	1	1	0.5	0.5	1	1	1	1	0.5	1	0.5	1	1	0.5	NA	1	1	1	1	1	1	1	21	87
Hor [28]	0	1	1	0.5	1	0.5	0	1	1	1	0	0.5	0	0.5	1	0.5	1	NA	0	0	1	1	0	0	1	13	54
Meng [37]	0.5	1	1	1	1	0.5	0.5	1	1	1	0.5	0.5	1	0.5	1	1	0.5	NA	1	1	1	1	1	1	1	20.5	85
Jespersen [29]	1	1	1	1	1	0.5	0.5	1	1	1	0.5	1	1	0.5	1	1	0.5	NA	1	1	1	1	1	1	1	21.5	90
Shida [30]	0.5	1	1	1	1	0.5	0.5	0.5	0	1	0	0.5	1	0.5	1	1	0.5	NA	1	0	1	1	1	1	1	17.5	73
Smith [38]	0.5	1	1	1	1	0.5	0.5	1	1	1	0.5	0.5	1	0.5	1	1	0.5	NA	1	1	1	1	1	1	1	20.5	85
West [39]	0.5	1	1	1	1	1	0.5	1	0	0	0	0.5	1	0.5	1	1	0.5	NA	1	0	1	1	1	1	1	17.5	73
Rizzardini [31]	1	1	1	1	1	0.5	0.5	1	1	1	0.5	0.5	1	0.5	1	1	0.5	NA	1	1	1	1	1	1	1	21	87
Davidson [32]	1	1	1	1	1	0.5	0.5	0.5	0	1	0.5	0.5	1	0.5	1	1	0.5	NA	1	1	1	1	1	1	1	19.5	81
Guillemard [33]	1	1	1	1	1	0.5	0.5	1	1	1	0.5	0.5	1	0.5	1	1	0.5	NA	1	0	1	1	0	0	1	18	75
Berggren [34]	1	1	0.5	1	1	0.5	0.5	0.5	0	0	0.5	0.5	1	0.5	1	1	0.5	NA	1	0	1	1	0	0	1	15	62
De Vrese [36]	1	1	1	1	0	0.5	0.5	0.5	1	1	0.5	0.5	1	0.5	1	1	0.5	NA	1	0	1	1	0	0	1	16.5	69
Tubelius [35]	1	1	1	1	1	0.5	0	0	0	1	0.5	0.5	0.5	0	1	0	0	NA	1	1	1	1	1	1	1	16	67

## Data Availability

Not applicable.
